# Identification of Pyrvinium, an Anthelmintic Drug, as a Novel Anti-Adipogenic Compound Based on the Gene Expression Microarray and Connectivity Map

**DOI:** 10.3390/molecules24132391

**Published:** 2019-06-28

**Authors:** Zonggui Wang, Zhong Dai, Zhicong Luo, Changqing Zuo

**Affiliations:** 1 Department of Biochemistry and Molecular Biology, Guangdong Medical University, Dongguan 523808, Guangdong, China; 2 Guangdong Key Laboratory for Research and Development of Natural Drugs, Zhanjiang 524023, Guangdong, China; 3 School of Pharmacy, Guangdong Medical University, Dongguan 523808, Guangdong, China

**Keywords:** pyrvinium, adipogenic differentiation, connectivity map, microarray

## Abstract

Obesity is a serious health problem, while the current anti-obesity drugs are not very effective. The Connectivity Map (C-Map), an in-silico drug screening approach based on gene expression profiles, has recently been indicated as a promising strategy for drug repositioning. In this study, we performed mRNA expression profile analysis using microarray technology and identified 435 differentially expressed genes (DEG) during adipogenesis in both C3H10T1/2 and 3T3-L1 cells. Then, DEG signature was uploaded into C-Map, and using pattern-matching methods we discovered that pyrvinium, a classical anthelminthic, is a novel anti-adipogenic differentiation agent. Pyrvinium suppressed adipogenic differentiation in a dose-dependent manner, as evidenced by Oil Red O staining and the mRNA levels of adipogenic markers. Furthermore, we identified that the inhibitory effect of pyrvinium was resulted primarily from the early stage of adipogenesis. Molecular studies showed that pyrvinium downregulated the expression of key transcription factors C/EBPa and PPARγ. The mRNA levels of notch target genes *Hes1* and *Hey1* were obviously reduced after pyrvinium treatment. Taken together, this study identified many differentially expressed genes involved in adipogenesis and demonstrated for the first time that pyrvinium is a novel anti-adipogenic compound for obesity therapy. Meanwhile, we provided a new strategy to explore potential anti-obesity drugs.

## 1. Introduction

Obesity is a serious global health problem of the recent several decades and it often represents an important risk factor for various chronic metabolic diseases, including type 2 diabetes, cardiovascular diseases, and cancers [1,2]. Obesity is often characterized by an increase in adipocyte number or adipocyte volume. The main reason for increase in the adipocyte number is the differentiation of mature adipocytes from mesenchymal stem cells or preadipocyte precursor cells (i.e., adipogenesis) [3]. Thus, inhibition of adipogenesis could be a useful approach for limiting obesity or achieving an anti-obesity effect.

Recent findings have revealed that adipogenesis is controlled by the sequential expression of many transcription factors. Among them, the two master regulators are peroxisome proliferator-activated receptor γ (PPARγ) and CCAAT/enhancer-binding protein a (C/EBPa), both of which are required and sufficient for adipocyte differentiation [4,5]. PPARγ and C/EBPa can mutually regulate each other and act cooperatively to induce the expression of adipogenesis-related genes, including the *FABP4* (422/aP2), *Adipsin*, and Adiponectin (*AdipoQ*), completing the adipocyte differentiation process [6].

Recently, bioinformatics and the systematic biology approach have made great contributions to new drug discovery [7]. The Connectivity Map (C-Map) database was developed in 2006 by the Broad Institute [8]. The C-Map database contains ~7000 whole-genome gene expression profiles from human cells treated with more than one thousand small molecules, together with pattern-matching software to mine these data. The C-Map database has provided the potential to reveal the connections among drugs, genes, and diseases. Recently, several studies, with the use of this method, have identified novel candidate therapeutic compounds to cancer [9,10], alzheimer’s disease [11], osteoporosis [12], etc. In particular, Junli Liu et al. have successfully used this approach to identify celastrol as a small molecule leptin sensitizer [13].

Pyrvinium pamoate is a classical anthelminthic officially approved by the FDA. Recently, it is attracting more and more attention as an anticancer drug though a variety of mechanisms [14] including: (a) inhibition of Akt and Wnt pathways [15,16], (b) suppression of the JAK2/STAT5 signaling pathway [17], and (c) attenuating Hedgehog signaling [18]. In addition, pyrvinium has also been reported to ameliorate myocardial contractile dysfunction [19] and inhibit osteogenic and chondrogenic differentiation of mesenchymal stem cells (MSCs) [20]. However, its effect on adipogenesis remains unexplored.

In this study, we identified pyrvinium as a novel anti-adipogenic differentiation agent using the C-Map database. Moreover, we verified that pyrvinium block adipogenesis through inhibiting notch signaling target genes such as *Hes1* and *Hey1*, leading to the down-regulated expression of the master regulators PPARγ and C/EBPa.

## 2. Results

### 2.1. Microarray Analysis Identifies Differential Expression of mRNAs during Adipogenesis in C3H10T1/2 and 3T3-L1 Cells

**Figure 1:**
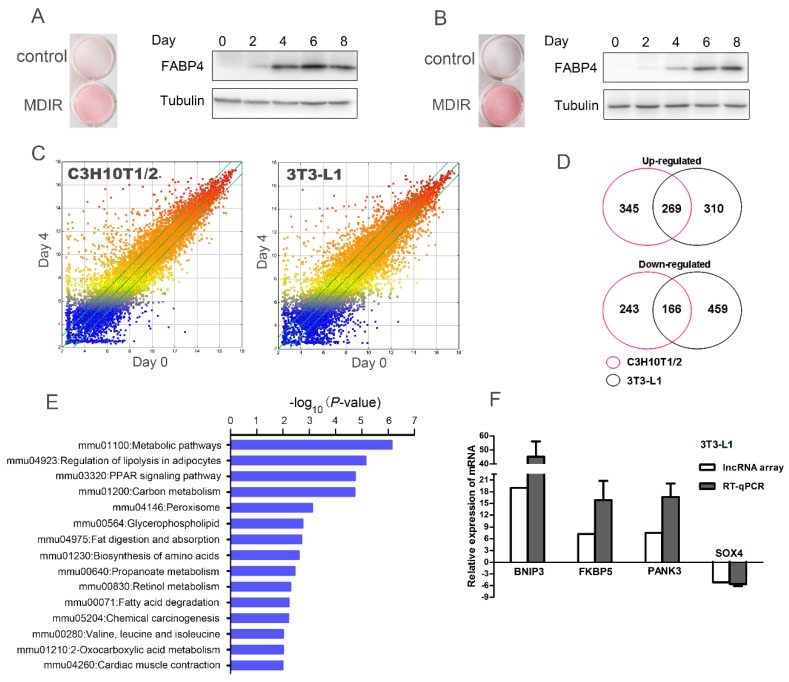
The differentially expressed genes in C3H10T1/2 and 3T3-L1 cells undergoing early adipocyte differentiation were identified. The Oil Red O staining and the protein expression of adipogenic marker FABP4 were analyzed after exposure of (**A**) C3H10T1/2 and (**B**) 3T3-L1 to differentiation inducers (IBMX, dexamethasone, insulin, and rosiglitazone, also called MDIR) for 8 days. (**C**) A scatterplot analysis was performed for assessing the variation between the samples. (**D**) The Venn diagram showed a list of more pertinent differentially expressed genes at day 0 and day 4 post-induction. (**E**) The Gene Ontology (GO) analysis and pathway analysis for the differentially expressed genes were conducted using DAVID web-based tools. (**F**) The expression tendency analysis of 4 randomly selected genes using lncRNA array and RT-qPCR demonstrated the reliability of the microarray data.

To obtain adipogenesis-related differential expression profiles of mRNAs, C3H10T1/2 mesenchymal stem cells (MSCs) and 3T3-L1 preadipocytes, two well-established cellular models of adipogenesis, were used as a model system. Exposure of C3H10T1/2 and 3T3-L1 to differentiation inducers (IBMX, dexamethasone, insulin, and rosiglitazone, also called MDIR) induced their differentiation into adipocytes, as evidenced by Oil Red O staining. Western blot analysis showed that the expression of FABP4, a typical adipocyte marker, was increased gradually after the induction of adipocyte differentiation (Figure 1A,B). To reveal the potential role of mRNAs at the early stage of adipogenesis, we used microarray to detect differentially expressed mRNAs on day 0 and day 4 post-induction. A scatterplot was used to assess the variation between the samples (Figure 1C). In total, after induction for 4 days, 1023 mRNAs (614 upregulated and 409 downregulated, fold change ≥ 4.0) and 1204 mRNAs (579 upregulated and 625 downregulated, fold change ≥ 4.0) were found to be significantly differentially expressed in C3H10T1/2 and 3T3-L1, respectively. To overcome the heterogeneity of gene expression profiles due to different cell lines and to obtain a list of more pertinent differentially expressed profiles for further study, the differentially expressed genes in both C3H10T1/2 and 3T3-L1 were designated as potential adipogenesis upregulated or downregulated genes. The Venn diagram showed that a total of 269 mRNAs were upregulated and 166 mRNAs were downregulated in both C3H10T1/2 and 3T3-L1 on day 4 post-induction (Figure 1D and Tables S1 and S2). Furthermore, Gene Ontology (GO) analysis and pathway analysis using DAVID web-based tools showed that the differentially expressed genes were mainly enriched in metabolic pathways, regulation of lipolysis in adipocytes, and the PPAR signaling pathway (Figure 1E). 

To validate the microarray data, we performed real-time quantitative PCR (RT-qPCR) analysis on four randomly selected differentially expressed mRNAs. The results showed that the expression tendency of 4 mRNAs were consistent with the microarray data (Figure 1F), which demonstrated the reliability of the microarray data.

### 2.2. Connectivity Map (C-Map) Analysis Identifies Pyrvinium as a Novel Anti-Adipogenic Compound

According to the Connectivity Map concept [8], a drug might exert an anti-adipogenic effect and treat obesity diseases if it could reverse the gene expression signature of adipogenesis. To identify the anti-adipogenic compounds using the C-Map, we performed the following analysis: First, we transformed potential adipogenesis upregulated or downregulated gene symbols to C-Map signatures (i.e., Affymetrix probe-sets, Tables S3 and S4). Second, we queried the C-Map database using C-Map signatures and obtained some compounds which reversed the signatures of adipogenesis. The top 10 compounds are summarized in Table 1. Most interestingly, the top two ranked compounds, tanespimycin (also named as 17-AAG) and LY-294002, have been proven very effective in preventing adipogenesis [21,22].

### 2.3. Pyrvinium Inhibits C3H10T1/2 and 3T3-L1 Adipogenesis in a Dose-Dependent Manner

**Figure 2:**
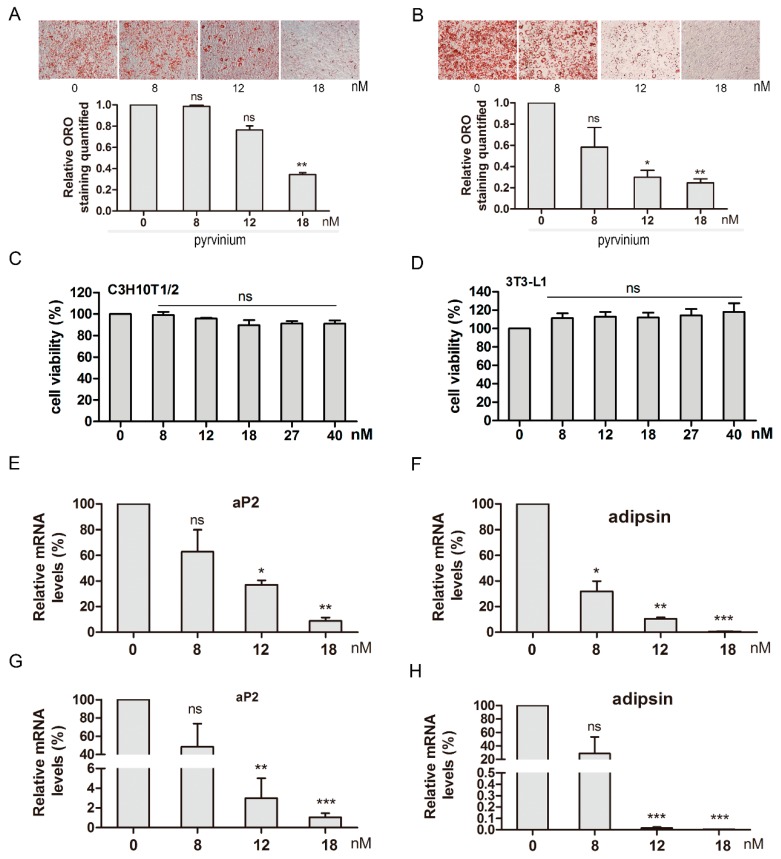
Pyrvinium inhibits adipocyte differentiation of C3H10T1/2 and 3T3-L1 cells. The Oil Red O (ORO) staining and relative quantification analysis at 8 days for adipogenic induction (**A**. C3H10T1/2, **B**. 3T3-L1). The cell viability analysis after pyrvinium treatment for 48h (**C**. C3H10T1/2, **D**. 3T3-L1). The mRNA expression of adipogenic marker *aP2* and *adipsin* after pyrvinium treatment (**E**,**F**. C3H10T1/2, **G**,**H**. 3T3-L1). The data are presented as mean ± SEM from three independent experiments. (* *p* < 0.05, * *p* < 0.01, * *p* < 0.001 compared with 0 nM).

Given that pyrvinium was ranked third among the candidate compounds using the C-Map screening method, we therefore selected it for further study. Treatment of C3H10T1/2 and 3T3-L1 cells with several concentrations of pyrvinium for 8 days resulted in dose-dependent inhibition of adipogenesis and lipid accumulation (Figure 2A,B). To exclude that the inhibition of adipogenesis and lipid accumulation by pyrvinium was due to cytotoxicity, we assessed its cytotoxicity using a CCK-8 assay after treatment for 48 h. However, no significant effects on cell viability in C3H10T1/2 and 3T3-L1 cells were observed at concentrations up to 40 nM (Figure 2C,D). Next, to further characterize the suppression effect of pyrvinium on adipogenic differentiation, we examined the mRNA expression of *Adipsin* and *aP2*, two markers for mature adipocytes. The results indicated that pyrvinium suppressed *Adipsin* and *aP2* mRNA levels in a dose-dependent manner (Figure 2E,H). Taken together, these results indicate that pyrvinium inhibits adipogenic differentiation.

### 2.4. Early Stage Adipogenic Differentiation is Critical for the Inhibitory Action of Pyrvinium

**Figure 3:**
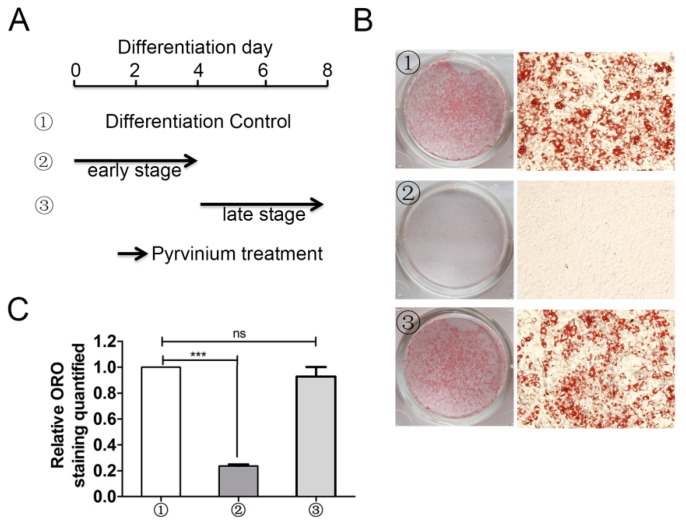
Anti-adipogenic effects of pyrvinium during the early stages of adipogenic differentiation. (**A**) A schematic model depicting pyrvinium treatment during C3H10T1/2 adipogenic differentiation. (**B**) Representative photographs of each treatment group after Oil Red O staining. C3H10T1/2 cells were treated with 18 nM pyrvinium for the indicated duration and cell monolayers were stained with Oil Red O after 8 days of adipogenic differentiation. (**C**) Spectrometric quantitative analysis of Oil Red O (ORO) staining. The stained adipocytes were extracted with isopropyl alcohol and the absorbance at 520 nm was measured. The data are presented as mean ± SEM from three independent experiments. (*** *p* < 0.001 compared with control).

Due to the critical role of anti-adipogenic differentiation for pyrvinium, we further elucidated the critical stage of adipogenic differentiation affected by pyrvinium treatment. The adipogenic differentiation was activated by sequential expression of transcription factors. Within 24–48 h following cell exposure to differentiation medium, the expression of C/EBPβ and C/EBPδ was transiently increased [23]. Then, the C/EBPa and PPARγ expression was gradually increased and maintained at a high level after 2 days. On approximately day 4, the adipocyte genes, such as fatty acid binding protein 4 (*FABP4*), began to increase and reached peak on about day 6–8, thereby leading to the adipocyte formation and maturation (Figure 1A,B). Based on the above, we arbitrarily divided the differentiation processes into two stages: the early stage (Day 0–4) and the late stage (Day 4–8). Then, C3H10T1/2 cells were treated with 18 nM pyrvinium at the early stage or late stage of adipogenesis. Compared with controls, as illustrated in Figure 3, the treatment group with pyrvinium at the early stage showed the significant inhibition of intracellular lipid droplet accumulation, whereas the treatment group at the late stage showed little inhibition of intracellular lipid accumulation. Collectively, these results indicate that pyrvinium exert has inhibitory effects at an early stage of adipogenic differentiation.

### 2.5. Pyrvinium Suppresses the Expression of Master Adipogenic Regulators

**Figure 4:**
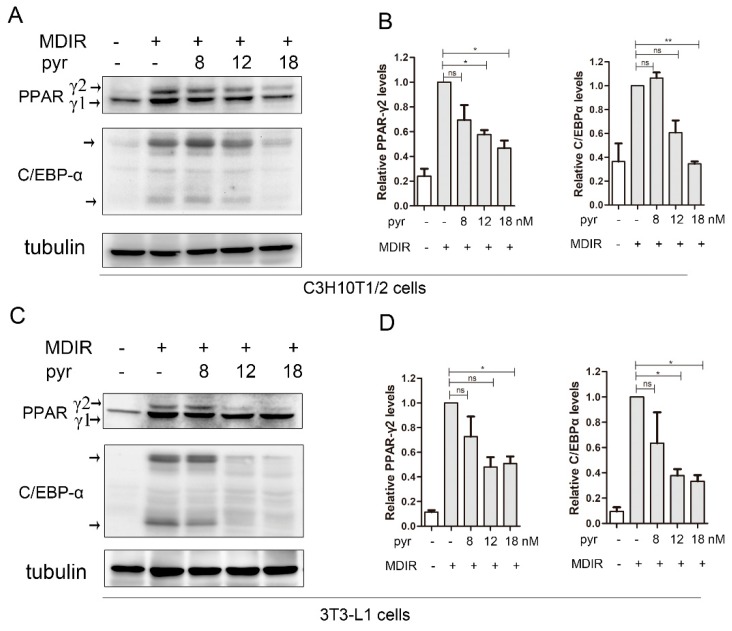
The effects of pyrvinium on the expression of adipogenic transcription factors. (**A**) The protein levels of PPARγ and C/EBPa were determined by western blot analysis after treatment with different doses of pyrvinium (pyr) for 3 days in C3H10T1/2 cells. (**B**) Western blot results in A were quantified against Tubulin. (**C**) The protein levels of PPARγ and C/EBPa were determined by western blot analysis after treatment with different doses of pyrvinium for 3 days in 3T3-L1 cells. (**D**) Western blot results in C were quantified against Tubulin. The data are presented as mean ± SEM from three independent experiments. (* *p* < 0.05, ** *p* < 0.01).

A series of specific transcription factors have been identified as regulators of adipogenesis. Among them, C/EBPa and PPARγ were considered as central transcriptional factors to maintain adipocyte-specific functions. To examine molecular mechanisms of inhibition adipogenesis by pyrvinium, we compared C/EBPa and PPARγ protein levels using western blot. Compared with the control cells, pyrvinium reduced the protein levels of C/EBPa and PPARγ in both C3H10T1/2 and 3T3-L1 cells in a dose-dependent manner (Figure 4).

### 2.6. Effect of Pyrvinium on the Notch Signaling Pathway during Adipogenic Differentiation

**Figure 5:**
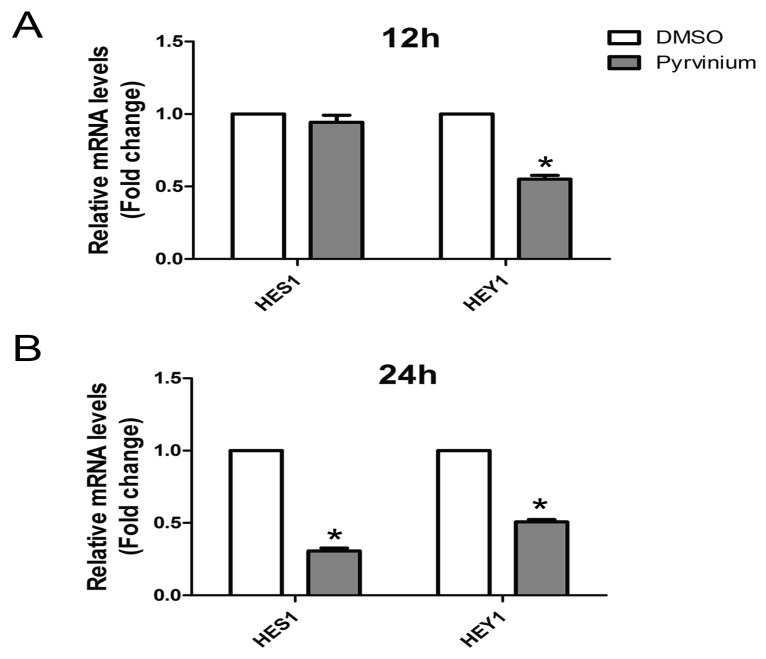
Pyrvinium suppresses the Notch signaling pathway during adipogenic differentiation. C3H10T1/2 cells were treated with DMSO or pyrvinium under adipogenic differentiation medium. Expressions of the Notch target genes *Hes1* and *Hey1* were measured by real-time PCR for 12 h (**A**) or 24 h (**B**). The data are presented as mean ± SEM from three independent experiments. Statistical significance was determined compared with control group (* *p* < 0.05).

Recently, Notch signaling was discovered as a positive modulator for adipogenesis. Notch inhibitor DAPT decreased the expression of PPARγ and C/EBPa [24], whereas the expression of PPARγ was enhanced after activation of Notch signaling [25]. Given that *Hes1* and *Hey1* are two most prominent targets of the Notch pathway, and to determine whether pyrvinium regulates the Notch pathway during adipogenic differentiation, we examined the expression of *Hes1* and *Hey1* by RT-qPCR analysis after pyrvinium treatment for 12 and 24 h. Compared with the control group, pyrvinium treatment resulted in a significant decrease of *Hes1* and *Hey1* mRNA levels (Figure 5). these results indicate that the Notch pathway plays an important role in inhibitory effects of pyrvinium for adipogenic differentiation.

## 3. Discussion

Adipogenesis is a complex biological process modulated by numerous intracellular and extracellular molecules. To date, the precise molecular mechanism of adipogenesis remains to be determined. New technologies such as microarrays facilitate for rapid identification of novel molecules involved in adipocyte regulation. In the current study, we identified a list of more pertinent differentially expressed genes for further study, which overcame the heterogeneity of gene expression profiles due to different cell lines. Meanwhile we also found the most enriched pathways in metabolic pathways, regulation of lipolysis in adipocytes, and the PPAR signaling pathway, thus supporting the notion that PPARγ plays a profound role in the initiation of adipogenic differentiation [26].

Many studies have elucidated therapeutic potentials of pyrvinium, an FDA approved anthelmintic drug, in cancer [14,18]. However, the effect of pyrvinium on adipogenesis or obesity is not clear yet. In the present study, we screened the candidate molecules involved in regulating adipocyte differentiation and found that pyrvinium was ranked third among candidate compounds using the C-Map method. Further research showed that pyrvinium, not due to its cytotoxicity, inhibited adipogenesis and lipid accumulation in a dose-dependent manner.

Over the last decade, multiple lines of evidence have demonstrated that the early stage of adipogenic differentiation is a very important and indispensable phase [3]. During the early stage, the adipogenesis-related transcription factors such as C/EBPβ, C/EBPa, and PPARγ were activated sequentially and cooperatively in the presence of hormonal inducers, which playing critical roles in mediating adipogenesis [5,27]. A series of new compounds, as potent inhibitors of the transcription factors activation, have been revealed with significant inhibitory effects on adipocyte differentiation [28,29,30]. In order to elucidate the critical stage of adipogenic differentiation affected by pyrvinium treatment, we arbitrarily divided the differentiation processes into the early stage (Day 0–4) and the late stage (Day 4–8). Consistent with those compounds reported previously [31,32], our data showed pyrvinium exerted its anti-adipogenic differentiation primarily in the early stages of differentiation. Furthermore, we observed the protein expressions of C/EBPa and PPARγ, two central transcriptional factors, were significantly diminished by pyrvinium treatment in both 3T3-L1 and C3H10T1/2 cells during adipocyte differentiation.

Pyrvinium was initially thought to be a pharmacologic inhibitor of Wnt and mainly blocking the Wnt/β-catenin pathway [33]. However, lots of evidence has demonstrated that Wnt inhibitors can promote preadipocyte differentiation by inhibiting Wnt/β-catenin signaling [34,35]. On the contrary, our results showed that pyrvinium could inhibit rather than promote adipocyte differentiation. We speculated that pyrvinium might affect adipocyte differentiation through other pathways. The recent evidence has also suggested that pyrvinium participated in a number of cellular processes other than Wnt signaling, such as Hedgehog signaling [18], and JAK2/STAT5 signaling [17]. The Notch signaling, an evolutionarily highly conserved signaling pathway, has an essential role in the regulation of cell proliferation and differentiation [36]. The Notch cascade mainly consists of Notch receptors, Notch ligands, and intracellular Notch target proteins such as Hes1 and Hey1. To date, many studies have elucidated that the Notch signaling pathway plays a role in adipocyte differentiation. For example, Notch inhibitors, including DAPT, compound E, and WPE-III-31C, can suppress adipogenic differentiation [24], whereas activation of Notch signaling by Jagged1 enhanced the adipogenesis of mASCs with the induction of PPARγ [25]. Interestingly, Notch target gene *Hes1* has dual roles in the differentiation of 3T3-L1 preadipocytes [37]. Given that *Hes1* and *Hey1* are two of the most prominent targets of the Notch pathway, which carry out Notch signaling functions, and to gain further insight into the mechanisms of anti-adipogenic effect of pyrvinium, we explored the expression levels of Hes1 and Hey1 with or without pyrvinium treatment at the early stage of adipogenic differentiation. Our data showed that pyrvinium treatment significantly reduced the expression levels of Hes1 and Hey1. Given that pyrvinium not only inhibits the expression of the key transcription factos C/EBPa and PPARγ but also suppresses the expression of Notch target *Hes1* and *Hey1*, and the Notch target gene can regulate the levels of C/EBPa and PPARγ [24,25,37], it is highly possible that pyrvinium modulate adipogenesis through suppression of the notch target *Hes1* and *Hey1*, maybe other unknown genes are involved, followed by down-regulated the expression of C/EBPa and PPARγ.

## 4. Materials and Methods

### 4.1. Cell Culture and Adipogenic Differentiation

The mouse C3H10T1/2 mesenchymal stem cells were provided by the Chinese Academy of Science Cell Bank (Shanghai, China) and 3T3-L1 preadipocytes were obtained from the Cell Resource Center of Peking Union Medical College (Beijing, China). C3H10T1/2 and 3T3-L1 cells were cultured in Dulbecco’s Modified Eagle’s Medium (DMEM; GIBCO) supplemented with 10% fetal bovine serum (FBS) or 10% newborn calf serum (NBCS), respectively. For adipogenic differentiation, cells were maintained in adipogenic medium containing 10% FBS, 500 µmol/L isobutylmethylxanthine (Sigma-Aldrich, I5879, Shanghai, China), 1 µmol/L dexamethasone (Sigma-Aldrich, D4902, Shanghai, China), 10 µg/mL insulin (Solarbio, I8040, Beijing, China), and 1 µmol/L rosiglitazone (Sigma-Aldrich, R2408, Shanghai, China) (also called MDIR), as previously reported [38]. To study the effect of pyrvinium on adipogenic differentiation, the cells were differentiated and supplemented with various concentrations of pyrvinium pamoate salt hydrate (Sigma-Aldrich, P0027, Shanghai, China), as indicated in the figures.

### 4.2. Oil Red O Staining

Eight days after adipogenic treatment in 12-well plates, the adherent cells were washed three times with PBS, and then fixed with 4% paraformaldehyde in PBS for 20 minutes. Subsequently, the Oil red O (ORO) staining and quantification were performed, as previously described [38]. Briefly, Oil Red O (ORO, Sigma) (0.5% in isopropanol) was diluted into 60% with water and incubated with the fixed cells for 30 min at room temperature. Then, cells were washed with PBS and photographed. To quantify the stained lipid content, ORO was extracted using 100% isopropanol for 5 min and the absorbance was measured at 520 nm.

### 4.3. Microarray Gene Expression Studies and Connectivity Map (C-Map) Analysis

To obtain the whole genome differentially expressed genes at the early stage of adipogenesis, we extracted total RNA from both C3H10T1/2 and 3T3-L1 cells at day 0 and day 4 post-induction for adipogenesis. The genome-wide profiling of mouse LncRNAs and protein-coding transcripts (mRNA) was detected by KangChen Bio-tech (Shanghai, China) using Arraystar Mouse LncRNA Microarray V3.0, which contains 35,923 LncRNAs and 24,881 coding transcripts (only differentially expressed mRNA was analyzed according to the C-Map database in this paper). The differentially expressed gene symbols were identified with a four-fold change cutoff for both C3H10T1/2 and 3T3-L1 cells. To overcome the effect of difference on cell types and to obtain consistent up-regulated or down-regulated gene symbols, we performed an analysis as follows: the up-regulated gene symbols in both C3H10T1/2 and 3T3-L1 cells were designated as potential adipogenesis up-regulated gene symbols, otherwise as potential adipogenesis down-regulated gene symbols. Then these gene symbols were converted to Affymetrix probe sets, which are required for the C-Map database analysis, using the Batch Query tools available at the Affymetrix website (https://www.affymetrix.com/analysis/index.affx). Lastly, these Affymetrix probe sets were submitted simultaneously to the C-Map (build02; www.broadinstitute.org/cmap/) for compounds screening. the Gene Ontology (GO) and pathway enrichment analysis of differentially expressed genes was performed using the Database for Annotation, Visualization, and Integrated Discovery (DAVID) web-based tools (https://david.ncifcrf.gov/) [39].

### 4.4. Cell Viability Assay

A Cell Counting Kit-8 (CCK-8, Dojindo, Kumamoto, Japan) assay was performed to examine cell viability. Briefly, cells were seeded in 96-well plates at 3000 cells per well and incubated overnight in normal growth medium. Then, the cells were treated with different concentrations of pyrvinium (0, 8, 12, 18, 27, and 40 nmol/L). After treatment for 48 h, cells were detected with the CCK-8 according to the manufacturer’s instructions.

### 4.5. RNA Preparation and Real-Time PCR Analysis

Total cellular RNA was isolated using Trizol (Life Technology, Rockville, MD, USA), and then the RNAs were reverse transcribed into cDNA using PrimeScript™ RT reagent Kit with gDNA Eraser (Takara Bio Inc., Otsu, Japan). Real-time PCR analysis was performed using SYBR^®^ PrimeScript™ RT-PCR Kit II (Takara Bio Inc., Otsu, Japan). 36B4 was used as an internal normalization control. The relative expression of mRNAs was calculated by the 2^−ΔΔCt^ method. All the primer sequences used for this study are shown in Supplementary Table S5.

### 4.6. Western Blot Analysis

Protein extractions were prepared with RIPA containing 1mM PMSF (Beyotime Institute of Biotechonogy, Haimen, China). Then the protein extractions were subjected to Western blot analysis, as previously described [38,40]. Antibodies to FABP4 (Santa Cruz Biotechnology, sc-271529, 1:1000), Antibodies to PPARγ (Santa Cruz Biotechnology, sc-7196, 1:200), Antibodies to C/EBPa (Cell Signaling Technologies, #8178, 1:1000), and Anti-Tubulin (Beyotime Biotechnology, AT819, 1:2000) were used as primary antibodies. After being incubated with HRP-labeled goat anti-mouse IgG (Beyotime Biotechonogy, A0216, 1:1000) or anti-rabit IgG (Beyotime Biotechonogy, A0208, 1:1000), the blots were visualized using electrochemiluminescence (ECL; Millipore, Darmstadt, Germany). The band signal intensities in the western blots were quantified by ImageJ software (https://imagej.nih.gov/ij/).

### 4.7. Statistical Analysis

With the exception of microarray experiments, all experiments were carried out with three biological replicates. The data were expressed as means ± SEM. The normality of the distribution of the results was evaluated using Shapiro–Wilk tests, and the homogeneity of variance was tested using Levene’s test. Differences between groups were analyzed using Student’s t-test, One-way analysis of variance, followed by Dunnett’s comparison test (for equal variance) or Dunnett’s T3 comparison test (for unequal variance), and *P* < 0.05 was taken as the statistical significance.

## 5. Conclusions

In summary, we discovered the pertinent expression profiles of mRNA for adipogenesis, which overcame the heterogeneity due to different cell lines. Our novel discovery of adipogenesis-related genes will lead to more studies and provides novel insight into the molecular mechanisms of adipogenesis in the future. Furthermore, through the C-Map in-silico drug screening approach, we found a list of the candidate anti-adipogenic compounds and identified pyrvinium-suppressed adipogenic differentiation through regulating the notch signaling pathway and inhibiting key transcription factors C/EBPa and PPARγ. These findings suggest that pyrvinium might be a novel therapeutic agent for obesity or obesity-related diseases therapy. Meanwhile, we provided a new strategy to explore potential anti-obesity drugs.

## Figures and Tables

**Table 1:** Connectivity Map (C-Map) permuted results showing the natural compounds with significant negative correlation to adipogenesis gene signatures.

Rank	C-Map Name	Mean C-Map Score ^1^	N ^2^	*p* Value
1	tanespimycin	−0.392	62	<0.00001
2	LY-294002	−0.241	61	0.00002
3	pyrvinium	−0.743	6	0.00048
4	prochlorperazine	−0.424	16	0.00170
5	terfenadine	−0.744	3	0.00172
6	morantel	−0.587	5	0.00256
7	triflusal	−0.806	3	0.00272
8	thioridazine	−0.441	20	0.00552
9	apigenin	−0.684	4	0.00577
10	Methylbenzethonium chloride	−0.515	6	0.00683

^1^ Ranging from +1 to −1, representing the enrichment scores about the query signature compared to each rank-ordered list in the C-Map database. ^2^ Sample numbers of related gene chips in the C-Map database.

## Supplementary Materials

The following are available online, Table S1: The up-regulated expression genes in C3H10T1/2 and 3T3-L1 cells during adipogenic differentiation, Table S2: The down-regulated expression genes in C3H10T1/2 and 3T3-L1 cells during adipogenic differentiation, Table S3: The transformed Affymetrix probe-sets from the up-regulated expression genes list, Table S4: The transformed Affymetrix probe-sets from the down-regulated expression genes list, Table S5: Primers used for RT-qPCR.
