# Supplementary material for: Identification of Pyrvinium, an Anthelmintic Drug, as a Novel Anti-Adipogenic Compound Based on the Gene Expression Microarray and Connectivity Map
Source: Molecules. 2019 Jun 28;24(13):2391. doi: 10.3390/molecules24132391 (PMC6650900; doi:10.3390/molecules24132391)
Supplement: Supplementary file 1 [file molecules-24-02391-s001.pdf]

# Supplementary materials

# Identification of Pyrvinium, an Anthelmintic Drug, as a Novel Anti-Adipogenic Compound Based on the Gene Expression Microarray and Connectivity Map

**Zonggui Wang [1], Zhong Dai [2,3], Zhicong Luo [2,3] and Changqing Zuo [2,3,*]**

1 Department of Biochemistry and Molecular Biology, Guangdong Medical University, Dongguan 523808, Guangdong, China
2 Guangdong Key Laboratory for Research and Development of Natural Drugs, Zhanjiang 524023, Guangdong, China
3 School of Pharmacy, Guangdong Medical University, Dongguan, Guangdong 523808, China
* Correspondence: zuochangqing@gdmu.edu.cn; Tel.: +86-769-22896551

**Table S1.** The up-regulated expression genes in C3H10T1/2 and 3T3-L1 cells during adipogenic differentiation.

| **Gene symbol/name** | | | | | | | | | | |
|---|---|---|---|---|---|---|---|---|---|---|
| Abca1 | Abhd5 | Acadvl | Aco2 | Acox1 | Acsf2 | Acsf3 | Acsl1 | Acss3 | Adh1 | Adhfe1 |
| Adig | Adipoq | Adrb2 | Adrb3 | Agpat2 | Agt | Aifm2 | Akr7a5 | Aldh1a1 | Aldh1a7 | Aldh3b2 |
| Alpl | Amacr | Angptl6 | Ankrd37 | Ano2 | Anxa8 | Apex2 | Apmap | Apol6 | Arhgap27 | Armc12 |
| Arxes1 | Arxes2 | Atp1a4 | Atp2b4 | Atp5g1 | B3gnt6 | BB287469 | Bckdhb | Bnip3 | Car5b | Ccdc103 |
| Ccdc125 | Ccdc69 | Cd160 | Ceacam15 | Ces1d | Ces1f | Cfd | Cidec | Cldn15 | Cluh | Clybl |
| Cma1 | Cmbl | Cox6a2 | Cox7a1 | Cpa1 | Crls1 | Cxcl13 | Cyc1 | Cycs | Cyp2b10 | Cyp2c44 |
| Cyp2c70 | Cyp2f2 | Defb43 | Dgat1 | Dgat2 | Dhrs3 | Dnajb9 | Ear1 | Ear10 | Ear2 | Ear3 |
| Ear6 | Ear7 | Echdc1 | Echs1 | Ehf | Ehhadh | Emc9 | Endog | Eppk1 | Ero1l | Fabp4 |
| Fabp5 | Fam13a | Fam178b | Fam195a | Fam213a | Fam214a | Fam83a | Fam89a | Fgf21 | Fitm2 | Fkbp5 |
| Folh1 | Fsd2 | G0s2 | Gbe1 | Gbgt1 | Gchfr | Gdf5 | Ghitm | Glul | Gm2016 | Gm2022 |
| Gm4794 | Gm8300 | Gnpat | Gpcpd1 | Gpd1 | Gpr81 | Gpt | Gpt2 | Gsdmc4 | Gys1 | Gys2 |
| Hibadh | Hibch | Higd1a | Hist1h1d | Hist1h4h | Hk2 | Hmha1 | Hp | Hpdl | Hsd11b1 | Idh3a |
| Ifngr1 | Itgae | Itpk1 | Ivns1abp | Kbtbd11 | Kbtbd13 | Kcnk15 | Kcnk3 | Klb | Krt36 | Krt79 |
| Larp1b | Lcn2 | Lin52 | Lipe | Lrrc39 | Map7d2 | Mapk6 | Mboat4 | Mc5r | Mcam | Mdh1 |
| Mettl7b | Mgst3 | Mical3 | Mpp3 | Mrap | Mreg | Msra | Mthfd2 | Mxi1 | Myc | Naaa |
| Nampt | Napsa | Nat1 | Niacr1 | Nr1h3 | Nrg4 | Nudt12 | Nudt17 | Nupr1 | Olr1 | Orm1 |
| Orm2 | Pank3 | Pcx | Pde3b | Pdss1 | Pex11a | Pfkfb3 | Pfkl | Phospho1 | Pim3 | Pkp1 |
| Pla2g12a | Pla2g16 | Plin1 | Pnpla2 | Por | Ppa1 | Ppargc1b | Ppp2r1b | Prg4 | Prrt4 | Psen2 |
| Psma1 | Pxmp2 | Rab15 | Rbmxl2 | Rcl1 | Rdh19 | Retn | Retnla | Rgcc | Rgn | Rprml |
| Rpusd3 | Scarb1 | Scp2 | Sdr9c7 | Selenbp1 | Selenbp2 | Serpina3c | Serpina3k | Sfxn1 | Sh2b2 | Sik2 |
| Slc13a4 | Slc16a10 | Slc1a5 | Slc22a20 | Slc25a10 | Slc25a19 | Slc25a34 | Slc2a4 | Slc36a2 | Slc5a6 | Smim5 |
| Smim6 | Sorbs1 | Sort1 | Srms | Strip2 | Syde2 | Syne1 | Taldo1 | Tead4 | Tgm2 | Thrsp |
| Tmem116 | Tmem216 | Tmem45b | Trim9 | Trpc1 | Tsc22d3 | Tst | Tusc5 | Txlng | Ubqln1 | Ucp3 |
| Ufsp1 | Usp49 | Vwa8 | Wnk4 | Zfp36l3 | 1600014C23Rik | 1700001J03Rik | 1700093K21Rik | 2010003K11Rik | 2610020H08Rik | 2610528A11Rik |
| 3010026O09Rik | 4632428N05Ri | 5730508B09Rik | 9430023L20Rik | A530016L24Rik | | | | | | |

**Table S2.** The down-regulated expression genes in C3H10T1/2 and 3T3-L1 cells during adipogenic differentiation.

| **Gene symbol/name** | | | | | | | | | | |
|---|---|---|---|---|---|---|---|---|---|---|
| Acta2 | Adamts5 | Adamts6 | Agtr2 | Ahr | Aif1 | Akap12 | Akr1c14 | Ankrd1 | Anxa3 | BC100451 |
| Bcl2 | Brs3 | C1rb | Cacnb2 | Casc4 | Ccl2 | Cd44 | Cdh11 | Ch25h | Chst2 | Cldn9 |
| Cmtm3 | Cnn2 | Col8a1 | Cp | Cpne8 | Csf1 | Csgalnact1 | Csprs | Cstad | Ctxn1 | Cxcl10 |
| Cxcl12 | Dab2 | Dact1 | Dclk1 | Dclk2 | Dcn | Ddah2 | Ddx60 | Dsel | Egr3 | Eln |
| Emp3 | Etv1 | Fam135b | Fam171a2 | Fam171b | Fam211b | Fap | Fat4 | Fgf7 | Fhl2 | Flrt2 |
| Flrt3 | Frmd4a | Fsip1 | Fstl1 | Gas7 | Gbp8 | Glt8d2 | Gm10406 | Gm13271 | Gm14446 | Gm4841 |
| Gm5077 | Gm5458 | Gm5886 | Gpr149 | Gpr176 | Grem1 | Gvin1 | Gxylt2 | Hgf | Hipk4 | I830012O16Rik |
| Ifi44 | Ifit2 | Igsf10 | Il1rapl1 | Isg15 | Islr | Itgav | Itgbl1 | Kalrn | Kcna4 | Kdm2b |
| Klhl38 | Lima1 | Lpcat2 | Mgp | Mlana | Mxra7 | Myl9 | Myo7a | Nov | Npy1r | Nrg1 |
| Nsun7 | Oas1a | Oas1h | Ogn | Pcdh7 | Pcdhga8 | Pdgfrb | Phlda1 | Pi15 | Plac8 | Plat |
| Plscr2 | Pmaip1 | Prrx1 | Ptger4 | Ptn | Ptplad2 | Ptx3 | Rai14 | Renbp | Rfpl4b | Rgs4 |
| Rnf157 | Rrad | Rspo3 | Rtp4 | Samd14 | Scg5 | Sh3kbp1 | Slc16a2 | Slc2a13 | Slc8a1 | Slit2 |
| Smarca1 | Socs5 | Sox4 | Sp100 | Spink5 | Swap70 | Sytl2 | Tgfb1i1 | Thbs1 | Thbs3 | Tiam2 |
| Tinag | Tlr2 | Tmem119 | Tmem173 | Tmem200a | Tnc | Tnfsf12 | Tnnt2 | Tpm1 | Trpm5 | Tspan6 |
| Ttn | Vgll3 | Vmn1r76 | Zc4h2 | Zfp287 | Zfp354c | Zfp521 | 1700026L06Rik | 2310030G06Rik | 4833427G06Rik | 5031410I06Rik |
| C530008M17Rik | | | | | | | | | | |

**Table S3.** The transformed Affymetrix probesets from the up-regulated expression genes list.

| Affymetrix U133A probesets | | | | | | | | | | |
|---|---|---|---|---|---|---|---|---|---|---|
| 200648_s_at | 200710_at | 200793_s_at | 200978_at | 201042_at | 201066_at | 201102_s_at | 201135_at | 201339_s_at | 201362_at | 201363_s_at |
| 201403_s_at | 201463_s_at | 201673_s_at | 201676_x_at | 201761_at | 201819_at | 201848_s_at | 201849_at | 201956_s_at | 201963_at | 202069_s_at |
| 202070_s_at | 202345_s_at | 202364_at | 202431_s_at | 202464_s_at | 202481_at | 202727_s_at | 202834_at | 202842_s_at | 202843_at | 202883_s_at |
| 202884_s_at | 202885_s_at | 202886_s_at | 202934_at | 202972_s_at | 202973_x_at | 203074_at | 203282_at | 203504_s_at | 203505_at | 203669_s_at |
| 203711_s_at | 203920_at | 203980_at | 204087_s_at | 204261_s_at | 204262_s_at | 204281_at | 204301_at | 204408_at | 204560_at | 204570_at |
| 204824_at | 204867_at | 204941_s_at | 204942_s_at | 204997_at | 205040_at | 205041_s_at | 205055_at | 205160_at | 205161_s_at | 205222_at |
| 205242_at | 205367_at | 205382_s_at | 205404_at | 205410_s_at | 205615_at | 205724_at | 205802_at | 205803_s_at | 205860_x_at | 205913_at |
| 205952_at | 205967_at | 206007_at | 206170_at | 206186_at | 206245_s_at | 206353_at | 206603_at | 206614_at | 206656_s_at | 206697_s_at |
| 206709_x_at | 206812_at | 207001_x_at | 207046_at | 207121_s_at | 207175_at | 207207_at | 207208_at | 207275_s_at | 207349_s_at | 207436_x_at |
| 207656_s_at | 207840_at | 208156_x_at | 208180_s_at | 208181_at | 208186_s_at | 208470_s_at | 208565_at | 208580_x_at | 208591_s_at | 208763_s_at |
| 208905_at | 208916_at | 208928_at | 208972_s_at | 209086_x_at | 209087_x_at | 209230_s_at | 209248_at | 209249_s_at | 209424_s_at | 209425_at |
| 209426_s_at | 209447_at | 209581_at | 209600_s_at | 209601_at | 209605_at | 209859_at | 210004_at | 210197_at | 210653_s_at | 210678_s_at |
| 210740_s_at | 210751_s_at | 210759_s_at | 210869_s_at | 211003_x_at | 211042_x_at | 211065_x_at | 211340_s_at | 211373_s_at | 211573_x_at | 211602_s_at |
| 211676_s_at | 211705_s_at | 211733_x_at | 211746_x_at | 211819_s_at | 212135_s_at | 212136_at | 212224_at | 212456_at | 212531_at | 212705_x_at |
| 212715_s_at | 212797_at | 212807_s_at | 212873_at | 212886_at | 212946_at | 213221_s_at | 213321_at | 213374_x_at | 213501_at | 213524_s_at |
| 213706_at | 213805_at | 213855_s_at | 213935_at | 214231_s_at | 214433_s_at | 214440_at | 214465_at | 214516_at | 214533_at | 214537_at |
| 214576_at | 214582_at | 214621_at | 214765_s_at | 215001_s_at | 215178_x_at | 215350_at | 215363_x_at | 215752_at | 215783_s_at | 215834_x_at |
| 215835_at | 216066_at | 216183_at | 217047_s_at | 217111_at | 217113_at | 217202_s_at | 217303_s_at | 217307_at | 217483_at | 217487_x_at |
| 217614_at | 217710_x_at | 217738_at | 217739_s_at | 217845_x_at | 217848_s_at | 218087_s_at | 218275_at | 218392_x_at | 218433_at | 218498_s_at |
| 218544_s_at | 218723_s_at | 218739_at | 218844_at | 219076_s_at | 219203_at | 219281_at | 219398_at | 219616_at | 219640_at | 219648_at |
| 219716_at | 219824_at | 219827_at | 219850_s_at | 219915_s_at | 219969_at | 219974_x_at | 220111_s_at | 220540_at | 220570_at | 220667_at |
| 220865_s_at | 221027_s_at | 221433_at | 221751_at | 221810_at | 221854_at | 221896_s_at | 222305_at | 222317_at | 222351_at | 32837_at |
| 39854_r_at | 41037_at | 59697_at | | | | | | | | |

**Table S4.** The transformed Affymetrix probesets from the down-regulated expression genes list.

| **Affymetrix U133A probesets** | | | | | | | | | | |
|---|---|---|---|---|---|---|---|---|---|---|
| 200974_at | 201058_s_at | 201107_s_at | 201108_s_at | 201109_s_at | 201110_s_at | 201278_at | 201279_s_at | 201280_s_at | 201416_at | 201417_at |
| 201418_s_at | 201605_x_at | 201645_at | 201860_s_at | 201893_x_at | 202052_s_at | 202191_s_at | 202192_s_at | 202262_x_at | 202273_at | 202291_s_at |
| 202351_at | 202820_at | 202863_at | 202864_s_at | 202949_s_at | 203666_at | 203684_s_at | 203685_at | 203729_at | 203873_at | 203874_s_at |
| 203875_at | 203889_at | 203921_at | 204285_s_at | 204286_s_at | 204337_at | 204338_s_at | 204339_s_at | 204358_s_at | 204359_at | 204462_s_at |
| 204489_s_at | 204490_s_at | 204501_at | 204533_at | 204802_at | 204803_s_at | 204846_at | 204896_s_at | 204897_at | 204924_at | 205185_at |
| 205399_at | 205422_s_at | 205440_s_at | 205483_s_at | 205534_at | 205535_s_at | 205611_at | 205635_at | 205717_x_at | 205782_at | 205991_s_at |
| 206029_at | 206078_at | 206115_at | 206116_s_at | 206117_at | 206157_at | 206237_s_at | 206343_s_at | 206426_at | 206427_s_at | 206501_x_at |
| 206617_s_at | 206673_at | 206932_at | 207004_at | 207005_s_at | 207053_at | 207082_at | 207172_s_at | 207173_x_at | 207191_s_at | 207248_at |
| 207293_s_at | 207294_at | 207369_at | 207374_at | 207704_s_at | 207776_s_at | 207823_s_at | 207938_at | 208127_s_at | 208189_s_at | 208195_at |
| 208230_s_at | 208231_at | 208232_x_at | 208241_at | 208408_at | 208475_at | 208476_s_at | 208782_at | 209079_x_at | 209108_at | 209109_s_at |
| 209306_s_at | 209307_at | 209335_at | 209369_at | 209465_x_at | 209466_x_at | 209561_at | 209647_s_at | 209648_x_at | 209651_at | 209687_at |
| 209716_at | 209835_x_at | 209897_s_at | 209901_x_at | 209955_s_at | 209960_at | 209961_s_at | 210218_s_at | 210219_at | 210273_at | 210368_at |
| 210369_at | 210517_s_at | 210557_x_at | 210755_at | 210757_x_at | 210804_x_at | 210872_x_at | 210916_s_at | 210941_at | 210985_s_at | 210986_s_at |
| 210987_x_at | 210997_at | 210998_s_at | 211066_x_at | 211067_s_at | 211103_at | 211104_s_at | 211737_x_at | 211805_s_at | 211813_x_at | 211839_s_at |
| 211896_s_at | 212014_x_at | 212063_at | 212509_s_at | 212670_at | 213095_x_at | 213665_at | 213668_s_at | 213714_at | 213866_at | 214059_at |
| 214321_at | 214453_s_at | 214587_at | 214635_at | 214909_s_at | 214927_at | 215051_x_at | 215294_s_at | 215303_at | 215365_at | 215389_s_at |
| 215537_x_at | 215775_at | 215787_at | 215836_s_at | 216005_at | 216056_at | 216269_s_at | 216598_s_at | 216724_at | 216725_at | 217053_x_at |
| 217061_s_at | 217502_at | 217523_at | 217892_s_at | 217996_at | 217997_at | 217998_at | 217999_s_at | 218000_s_at | 218468_s_at | 218469_at |
| 218730_s_at | 218986_s_at | 219014_at | 219049_at | 219179_at | 219250_s_at | 219427_at | 219684_at | 219935_at | 219950_s_at | 220040_x_at |
| 220194_at | 220327_at | 220613_s_at | 220663_at | 220866_at | 221152_at | 221447_s_at | 221910_at | 221911_at | 222321_at | 33197_at |

**Table S5.** Primers used for RT-qPCR.

| Gene name | Forward primer (5'--3') | Reverse primer (5'--3') |
|---|---|---|
| aP2 | GATGCCTTTGTGGGAACCTG | CAGTTTGAAGGAAATCTCGGT |
| Adipsin | CTGAACCCTACAAGCGATGG | ACCCAACGAGGCATTCTG |
| Hes1 | TGCCAGCTGATATAATGGAGAA | CCATGATAGGCTTTGATGACTTT |
| Hey1 | CATGAAGAGAGCTCACCCAGA | GAACACAGCGCCGAACTCAA |
| Bnip3 | TGGATGCCCAGCATGAATCTG | CTGTGGCTGTCTATTTCAGCTC |
| Fkbp5 | TATGCTTATGGCTCGGCTGG | GAGTATCCCTCGCCTTTCCG |
| Pank3 | GGTGCTGAGTGCTACAGGAG | GCAAGCCCTTTACAAGGCAG |
| Sox4 | GGACAGCGACAAGATTCCGT | TGCCCGACTTCACCTTCTTTC |
| 36B4 | GCTTCATTGTGGGAGCAGAC | ATGGTGTTCTTGCCCATCAG |
